# Using lncRNA Sequencing to Reveal a Putative lncRNA-mRNA Correlation Network and the Potential Role of PCBP1-AS1 in the Pathogenesis of Cervical Cancer

**DOI:** 10.3389/fonc.2021.634732

**Published:** 2021-03-23

**Authors:** Linhan Li, Qisong Peng, Min Gong, Ling Ling, Yingxue Xu, Qiaoling Liu

**Affiliations:** ^1^ Department of Gynaecology and Obstetrics, Affiliated Jiangning Hospital of Nanjing Medical University, Nanjing, China; ^2^ Department of Clinical Laboratory, Affiliated Jiangning Hospital of Nanjing Medical University, Nanjing, China

**Keywords:** lncRNA-mRNA correlation network, long non-coding RNA, cervical cancer, lncRNA sequencing, proliferation

## Abstract

**Background/Aims:**

Long non-coding RNAs (lncRNAs) play important roles in many diseases and participate in posttranscriptional regulatory networks in tumors. However, the functions of major lncRNAs in cervical cancer are unclear. Therefore, the aim of this study was to construct a lncRNA-mRNA coexpression functional network and analyze lncRNAs that might contribute to the pathogenesis of cervical cancer.

**Methods:**

Differentially expressed lncRNAs (DElncRNAs) and mRNAs (DEmRNAs) between three pairs of cervical cancer tissues and adjacent mucosa were identified by lncRNA microarray analysis. LncRNA-mRNA correlation analysis and functional enrichment were performed on the DEGs. From the correlation network, PCBP1-AS1 was selected as a candidate for further analysis. PCBP1-AS1 expression was examined by qPCR, and Kaplan–Meier survival, clinicopathology, GSEA, and immune infiltration analysis of PCBP1-AS1 were performed. The immune responses of PCBP1-AS1 expression in cervical cancer were analyzed using TIMER and western blot. PCBP1-AS1 was knocked down and overexpressed to evaluate its role in cell proliferation, migration, and invasion.

**Results:**

A total of 130 lncRNAs were significantly differentially expressed in cervical cancer patient samples compared with control samples. Differentially expressed mRNAs in the lncRNA-mRNA interaction network were involved in the EMT process. Combined with the Kaplan–Meier survival analyses, the coexpression network revealed that PCBP1-AS1 was significantly associated with OS and clinicopathological parameters in cervical cancer patients. Moreover, PCBP1-AS1 expression was not only significantly increased in cervical cancer specimens but also associated with tumor stage, TNM, and invasion. GSEA revealed that PCBP1-AS1 is closely correlated with cell biological function *via* the p53 and notch signaling pathways. TIMER analysis revealed that the numbers of NK cells and M2 macrophages decreased when PCBP1-AS1 expression was high, which was consistent with the western blot results in clinical samples. Furthermore, *in vitro* experiments showed that high expression of PCBP1-AS1 promoted cell proliferation, migration, and invasion.

**Conclusions:**

Transcriptomic and lncRNA-mRNA correlation analyses revealed that PCBP1-AS1 plays a key role as an independent prognostic factor in patients with cervical cancer. The identification of PCBP1-AS1 as a new biomarker for cervical cancer could help explain how changes in the immune environment promote cervical cancer development.

## Introduction

Cervical cancer is one of the most common malignancies in female patients, and it has the highest mortality of all female reproductive system malignancies (1, 2). Moreover, its prevalence rate is rising among young women (3). Most patients suffering from cervical cancer are diagnosed at advanced stages, accompanied by invasion and distant metastasis (4, 5). At present, surgical resection and chemotherapy are the first-tier options of cervical cancer treatments, but tumor metastasis and recurrence still lead to poor prognosis (5–7). Although some progress has been made in research on the mechanism of cervical cancer, clinical applications are still limited, resulting in persistently high mortality in cervical cancer (8, 9). Therefore, the discovery of new mechanisms associated with cervical cancer for the identification of useful biomarkers as well as new specific therapeutic targets in cervical cancer is urgently needed.

In recent years, high-throughput transcriptome sequencing has become very common, revealing that up to 70% of the human genome is transcribed. However, the coding-protein transcripts are less than 2%, and most transcripts belong to non-protein-coding RNAs (ncRNAs) (10), including microRNAs (miRNAs), long non-coding RNAs (lncRNAs), and circular RNAs. Accumulating evidence has demonstrated that ncRNAs play crucial roles in the occurrence and progression of tumors (11–13). In the past decade, lncRNAs, defined as transcripts with a length of more than 200 nt, have been found to play key roles in multiple types of human tumorigenesis, metastasis, and chemotherapy resistance (14–16). Nevertheless, most of the functional lncRNAs in cervical cancer have yet to be identified. The mechanism of lncRNA function is associated with its target mRNAs. Therefore, lncRNA induced target mRNA transcription disorders was an effective strategy to identify key functional lncRNAs for cancer. For example, to search for candidate prostate cancer-related lncRNAs, lncRNA-mRNA bipartite networks, and lncRNA-mRNA coexpression networks have been constructed (17, 18). Although the mechanism of lncRNAs is not fully understood, they have already been considered potential biomarkers and therapeutic targets for many tumors (19, 20).

Here, we performed lncRNA-seq to investigate the expression levels of lncRNAs and mRNAs in six cervical cancer samples (three paired cervical cancer and adjacent mucosa) and constructed a lncRNA-mRNA coexpression network to identify the role of the candidate lncRNAs in the expression, prognosis, clinical pathology, immune infiltration, proliferation, migration, and invasion of cervical cancer and HeLa cells.

## Materials and Methods

### Sample Collection and Preparation

A total of three pairs of cervical cancer and adjacent tissues were collected from three cervical cancer patients who underwent surgical operation from July 2019 to August 2012 in Department of Gynecology and Obstetrics, Affiliated Jiangning Hospital of Nanjing Medical University. After sequencing, we collected another 20 pairs of samples for data verification (**Supplementary Table 1**). Among them, 15 pairs (15 cervical cancer tissues and 15 controls) were collected from June 2020 to December 2020 for qPCR assay. Another five pairs of samples (five cervical cancer tissues and five controls) were collected between January 2021 and February 2021 to indirectly measure the number of immune cells in cervical cancer samples.

Specimens were frozen in liquid nitrogen immediately after operation and stored at −80°C until extraction. All samples were confirmed by histopathological examination. This study was approved by the ethics committee of the hospital. Informed consent to collection and use of the biological samples was obtained from each patient.

### LncRNA Microarray

Total RNA was isolated using a RNeasy mini kit (Qiagen, Germany) and analyzed by 1% agarose gel electrophoresis (Bio-Rad, USA) to ensure that no degradation occurred. The RNA libraries were constructed using the TruSeq RNA Sample Preparation Kit (Illumina, USA). After purification, libraries were quantified using a Qubit 8000 (Life Technologies, USA) and validated with an Agilent 2100 (Agilent Technologies, USA) to confirm the insert size. Then, clusters were sequenced on an Illumina HiSeq 2500 instrument (Illumina, USA). Library construction and sequencing were performed at Shanghai Yuanshen Biomedical Technology Co., Ltd.

### Data Analysis

Differentially expressed lncRNAs and mRNAs were identified through fold-change filtering (|log2FC|>1 and p<0.05) using the “edgeR” package in R. The differentially expressed RNA profiles were normalized by log2 transformation.

### LncRNA-mRNA Correlation Network

The Pearson correlation coefficient was calculated, and the R value (cutoff >0.95) was used for each pair of lncRNA-mRNA interactions. The lncRNA-mRNA correlation network was constructed by Cytoscape software.

### GO and KEGG Functional Enrichment Analysis

Functional enrichment analysis of lncRNA-target mRNAs was performed using Metascape (https://metascape.org/). All statistically enriched terms (Gene Ontology and Kyoto Encyclopedia of Genes and Genomes) were identified based on accumulative hypergeometric *p* values.

### Kaplan–Meier Survival Analysis of lncRNAs

To investigate the predictive value of the expression levels of lncRNAs and mRNAs for the survival of cervical cancer patients, Kaplan–Meier survival analysis was performed using GEPIA (http://gepia2.cancer-pku.cn/). The statistical significance was set at *p* < 0.05. Then, the data obtained from the analysis were verified by StarBase (http://starbase.sysu.edu.cn/).

### Cox Proportional Regression Model Based on Differentially Expressed RNAs

To analyze the independent effects of individual miRNAs on the overall survival of patients with colon cancer, we performed univariate and multivariate Cox proportional regression analysis with an online tool (SangerBox tools, http://sangerbox.com/Tool). We constructed a Cox proportional hazards regression model and calculated the risk value of each patient through the formula (risk score = b × exp (RNA1) + b × exp (RNA2) +… + b × exp (RNAn), where b represents the multivariate Cox regression coefficient and exp () represents the expression level of prognostic RNAs. Next, we calculated the survival rates of the high-risk and low-risk groups and plotted the 1-year, 3-year, and 5-year survival receiver operating characteristic (ROC) curves to test the feasibility of the prediction ability of the model.

### RNA Extraction and Quantitative PCR

Total RNA was extracted from cervical cancer samples using an RNeasy mini kit (Qiagen, Germany) and analyzed by 1% agarose gel electrophoresis (Bio-Rad, USA) to ensure that no degradation had occurred. A Qubit 8000 (Life Technologies, USA) was used to measure the RNA concentration. Then, the RNA was reverse transcribed into cDNA. qRT-PCR was performed using SYBR Premix Ex Taq (Takara, China) on an ABI7500 system (Applied Biosystems, CA). The following cycling parameters were used: initial denaturation at 95°C for 30 s, followed by 35 cycles of 95°C for 5 s, 58°C for 30 s and 95°C for 60 s, and 60°C for 30 s. The primer sequences for PCR were as follows: PCBP1-AS1, forward: 5′-CCAACCTGATACATTGCCT-3′ and reverse 5′-TGGAAGAAATTCCCTGCTG-3′, GAPDH: forward 5′-CTCCTCCACCTTTGACGCTG-3′ and reverse 5′-TCCTCTTGTGCTCTTGCTGG-3′. Primers were synthesized by Sangon Biotech (China). GAPDH was used as a control. The mean value of triplicate experiments was used to calculate relative lncRNA expression using the formula ΔCt = Ct^mean^ lncRNAs − Ct^mean^ GAPDH. Expression fold changes were calculated using the 2-^ΔΔCt^ method.

### Immune Infiltrates Analysis

The TIMER (https://cistrome.shinyapps.io/timer/) correlation module was used to evaluate potential relationships between PCBP1-AS1 expression and immune infiltrates.

### Gene Set Enrichment Analysis

GSEA was performed using normalized RNA-Seq data obtained from TCGA-cervical cancer. The number of permutations was set to 100. Using GSEA, we further analyzed GO terms and KEGG pathways to investigate possible biological functions of PCBP1-AS1 (p-value <0.05).

### Cell Culture

HeLa cells were obtained from the American Type Culture Collection (ATCC). The cell lines were cultured as suggested by ATCC. The cells were cultured in Dulbecco’s modified Eagle’s medium (Invitrogen, USA) supplemented with 10% fetal bovine serum (Invitrogen, USA), 100 U/ml penicillin (Sigma, USA), and 100 µg/ml streptomycin (Sigma, USA) under a humidified atmosphere of 5% CO_2_ at 37°C.

### Cell Transfection

PCBP1-AS1 small interfering RNA (si-PCBP1-AS1) and the corresponding control (si-NC) were purchased from RiboBio (Guangzhou, China). The PCBP1-AS1 overexpression plasmid (pCDH-GFP-PCBP1-AS1) and corresponding control plasmid (NC) were also purchased from RiboBio (Guangzhou, China). All oligomers and plasmids were transfected into HeLa cells using Lipofectamine 3000 reagents (Invitrogen, USA) based on the manufacturer’s protocol. Briefly, when HeLa cell densities were approximately 60% in 12-well plates (Corning, USA), 50 nM siRNA oligos or 2 µg overexpression plasmids were introduced into cells using Lipofectamine 3000 reagents (Invitrogen, USA). Untreated cells were set as blank groups and transfected with empty vectors, and NC-siRNA was used as a negative control. At 24 h post transfection, the efficiency of knockdown and overexpression was determined by qRT-PCR and fluorescence microscopy. Subsequent experiments were performed at 48 h after transfection.

### Proliferation Assays

The proliferation of HeLa cells was measured by cell proliferation using Cell Counting Kit-8 (Sigma, USA) in 96-well plates. Then, 3,000 cells/well were incubated for 12, 24, and 48 h. All cells were then incubated with CCK-8 reagent (10 μl per well) for 3 h, and a microplate reader (Thermo, USA) was utilized to detect the absorbance of each well at 450 nm. Each experiment was carried out three times.

### Wound‐Healing Assay

HeLa cells were seeded in plates (96 wells, Corning, USA) at 5 × 10^5^ cells/well with culture medium at 37°C with 5% CO_2_. Then, the confluent cell monolayer was scratched with a sterile 200 μl pipette tip, and Opti-MEM™-reduced serum medium (Gibco, USA) was added. Microscope photos were taken after 0, 12, 24, and 48 h to record the scratched areas. ImageJ software was used to evaluate the percentage of closure.

### Transwell Assay

At 48 h after transfection, HeLa cells were collected to prepare a single-cell suspension. The HeLa cell suspension (3 × 10^3^ cells/well) was added to the Transwell upper chamber (Corning, USA), and DMEM (20% FBS) was added to 24-well plates in the lower chamber. The upper chamber was coated with Matrigel. After 24 h, 4% paraformaldehyde (Sigma, USA) was applied to fix the cells, and the cells were stained with 1% crystal violet (Sigma, USA). Cells were observed and counted under an optical microscope (Olympus, Japan).

### Western Blot Analysis

Total protein was extracted using RIPA lysis buffer (Beyotime, China), and separated using 10% SDS-PAGE (Beyotime, China). Next, proteins were transferred onto PVDF membranes (Beyotime, China). Then, the target protein membrane was blocked with 5% nonfat milk for 24 h. Subsequently, the membranes were incubated with specific primary antibodies against CD4 (Beyotime, China, 1:1,000), CD19 (Beyotime, China, 1:1,000), CD56 (Beyotime, China, 1:1,000), and GAPDH (Beyotime, China, 1:1,000) for 24 h at 4°C. GAPDH was used as a control. Afterwards, the membranes were incubated with secondary antibodies (Beyotime, China, 1:1,000) for 4 h at room temperature. TMB color liquid (Beyotime, China) was used to detect protein bands. ImageJ software was used to analyze the gray value of the bands. Protein levels were calculated using the ratio of target protein/GAPDH.

### Statistical Analysis

GraphPad Prism 8.0 software (California, USA) was utilized to perform statistical analysis. The discrepancies between two groups were compared by t-test. The differences were deemed statistically significant at p < 0.05.

## Result

### Differential Expression Patterns of Genes Between Cervical Cancer Tissues and Adjacent Tissues

**Figure 1:**
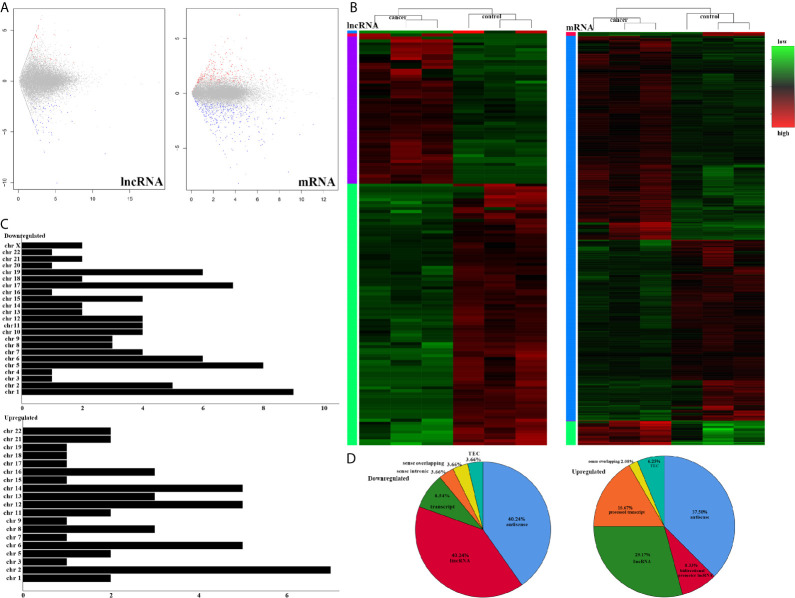
LncRNA sequencing of CESC. **(A)** Volcano plots of differential expression profiles of lncRNAs and mRNAs. The red dots represent upregulated genes. The blue dots represent downregulated genes. The gray dots represent genes that do not differ significantly. **(B)** Heat map of differential expression profiles of lncRNAs and mRNAs. **(C)** Chromosomal distribution of all differentially expressed lncRNAs. **(D)** Fraction distribution of all category-annotated DEG long non-coding RNAs (lncRNAs).

To understand the lncRNAs and mRNAs involved in cervical cancer pathogenesis, we performed lncRNA microarray detection in three pairs of cervical cancer tissues and matched adjacent tissues. On average, 82.58 million reads were obtained for each sample (**Supplementary Table 2**). Among the 10,675 detected expressed lncRNAs, we identified 130 differentially expressed lncRNAs between cervical cancer tissues and adjacent tissues (|log2FC|>1 and *p* < 0.05), of which 48 were upregulated and 82 were downregulated in the cancer tissue, as shown in **Figure 1A**; the red points represent statistically significant upregulated differentially expressed lncRNAs, and the blue points represent downregulated differentially expressed lncRNAs. With this same criterion, we identified 656 significantly differentially expressed mRNAs, of which 293 were upregulated and 363 were downregulated (**Figure 1A**). The top 10 lncRNAs (up- and down) and mRNAs (up- and down) are shown in **Tables 1** and **2**, respectively. Among the annotated differentially expressed lncRNAs, antisense lncRNAs accounted for 37.50% of upregulated lncRNAs, whereas the majority of downregulated lncRNAs were lincRNAs and antisense lncRNAs (accounting for 40.24% each) (**Figure 1D**). The distribution of differentially expressed lncRNAs across chromosomes was also analyzed. Among the downregulated differentially expressed lncRNAs, chromosome 1 had the most differentially expressed lncRNAs (n = 9), followed by chromosome 5 (n = 8) (**Figure 1C**). In the same analysis, chromosome 2 had the most DElncRNAs (n = 7) among the upregulated differentially expressed lncRNAs (**Figure 1C**). The heat map results in **Figure 1B** show that lncRNA and mRNA expression was distinct between cervical cancer tissues and adjacent tissues. In summary, the results from the lncRNA microarray analysis indicated that aberrantly expressed genes, including mRNAs and lncRNAs, may play important roles in the development and progression of cervical cancer.

**Table 1:** Top10 (up- and downregulated) of DElncRNAs in normal tissues and CESC tissues.

ID	Symbol	lncRNA type	logFC	padj	chr	P Value
**Upregulation**						
ENSG00000253339	AC111149.2	lincRNA	12.68869	0.040587	8	0.000396
ENSG00000257588	AC025154.2	antisense	12.06594	0.000559	12	1.27E-06
ENSG00000235954	TTC28-AS1	processed_transcript	11.94252	0.046726	22	0.00051
ENSG00000236778	INTS6-AS1	antisense	11.15821	0.000319	13	5.51E-07
ENSG00000258592	AL391152.1	lincRNA	11.14377	0.034594	14	0.000323
ENSG00000248092	NNT-AS1	antisense	11.08669	0.001126	5	3.09E-06
ENSG00000232940	HCG25	antisense	10.29039	0.01226	6	7.89E-05
ENSG00000179818	PCBP1-AS1	processed_transcript	10.18153	0.003325	2	1.23E-05
ENSG00000231074	HCG18	antisense	10.17907	2.23E-05	6	2.62E-08
ENSG00000232306	AC012485.2	lincRNA	10.12062	0.004252	2	1.88E-05
**Downregulation**						
ENSG00000231062	AC103563.2	antisense	−13.6321	1.03E-07	2	2.42E-11
ENSG00000251562	MALAT1	lincRNA	−13.0168	2.33E-13	11	3.65E-17
ENSG00000285756	BX890604.2	lincRNA	−12.4793	0.000115	X	1.62E-07
ENSG00000203688	LINC02487	lincRNA	−12.4505	1.02E-05	6	9.64E-09
ENSG00000228789	HCG22	lincRNA	−11.7357	3.20E-05	6	4.26E-08
ENSG00000215458	AATBC	antisense	−11.6164	0.043124	21	0.000454
ENSG00000250167	AC034206.1	antisense	−11.3856	0.000559	5	1.23E-06
ENSG00000237499	AL357060.1	antisense	−11.3592	0.003325	6	1.25E-05
ENSG00000266729	DSG1-AS1	antisense	−11.1001	0.003806	18	1.55E-05
ENSG00000279717	AC005336.3	TEC	−10.8012	8.72E-06	19	7.29E-09

**Table 2:** Top 10 (up- and downregulated) of DEmRNAs in normal tissues and CESC tissues.

ID	Symbol	Gene type	logFC	padj	Chr	PValue
**Upregulation**						
ENSG00000124208	TMEM189-UBE2V1	pc	10.95920703	0.028272279	20	0.000949057
ENSG00000162896	PIGR	pc	8.320731276	0.002015361	1	2.45E-05
ENSG00000157765	SLC34A2	pc	7.449877017	2.22E-10	4	1.32E-13
ENSG00000131152	AC010531.1	pc	7.366463382	0.000672218	16	5.55E-06
ENSG00000187908	DMBT1	pc	7.339636292	4.89E-05	10	2.34E-07
ENSG00000169064	ZBBX	pc	7.131696411	0.0001358	3	8.59E-07
ENSG00000083782	EPYC	pc	7.062597097	0.041539105	12	0.001665568
ENSG00000173702	MUC13	pc	6.797754334	0.001353022	3	1.42E-05
ENSG00000117983	MUC5B	pc	6.19862045	0.014474512	11	0.000361908
ENSG00000047457	CP	pc	6.149951455	3.30E-24	3	7.54E-28
**Downregulation**						
ENSG00000124766	SOX4	pc	1.065906521	0.005626318	6	9.64E-05
ENSG00000205593	DENND6B	pc	1.062334084	0.026703654	22	0.000882663
ENSG00000163902	RPN1	pc	1.061712008	0.042212725	3	0.001717434
ENSG00000075420	FNDC3B	pc	1.044223744	0.040616111	3	0.001619221
ENSG00000100629	CEP128	pc	1.039944887	0.045860025	14	0.00195825
ENSG00000166762	CATSPER2	pc	1.033277218	0.046291255	15	0.002005818
ENSG00000147400	CETN2	pc	1.01823592	0.025777184	X	0.000842572
ENSG00000162065	TBC1D24	pc	1.013031342	0.000746529	16	6.39E-06
ENSG00000125148	MT2A	pc	1.012283062	7.62E-05	16	3.93E-07
ENSG00000118707	TGIF2	pc	1.008808568	0.037527843	20	0.001411017

### lncRNA-RNA Interaction Network and GO Analysis

**Figure 2:**
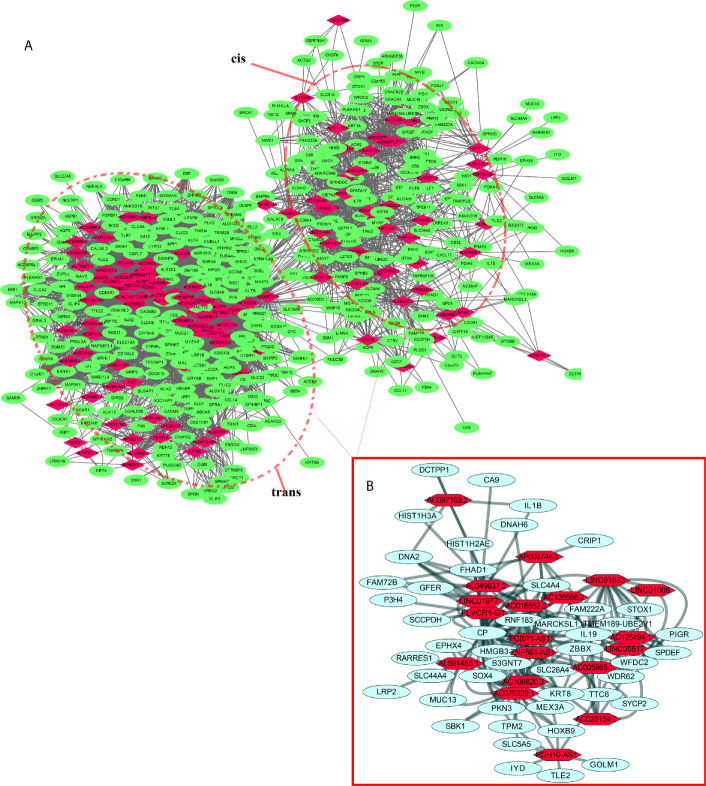
LncRNA-mRNA interaction network. **(A)** Correlation analysis was carried out with differentially expressed lncRNAs and mRNAs, and the cutoff value was set with a threshold of correlation >0.95. Red nodes represent lncRNAs, green nodes represent mRNAs, and red dotted boxes indicate trans and cis groups. **(B)** The red solid line box indicates the highly active subnetwork module identified by the jActive module.

**Figure 3:**
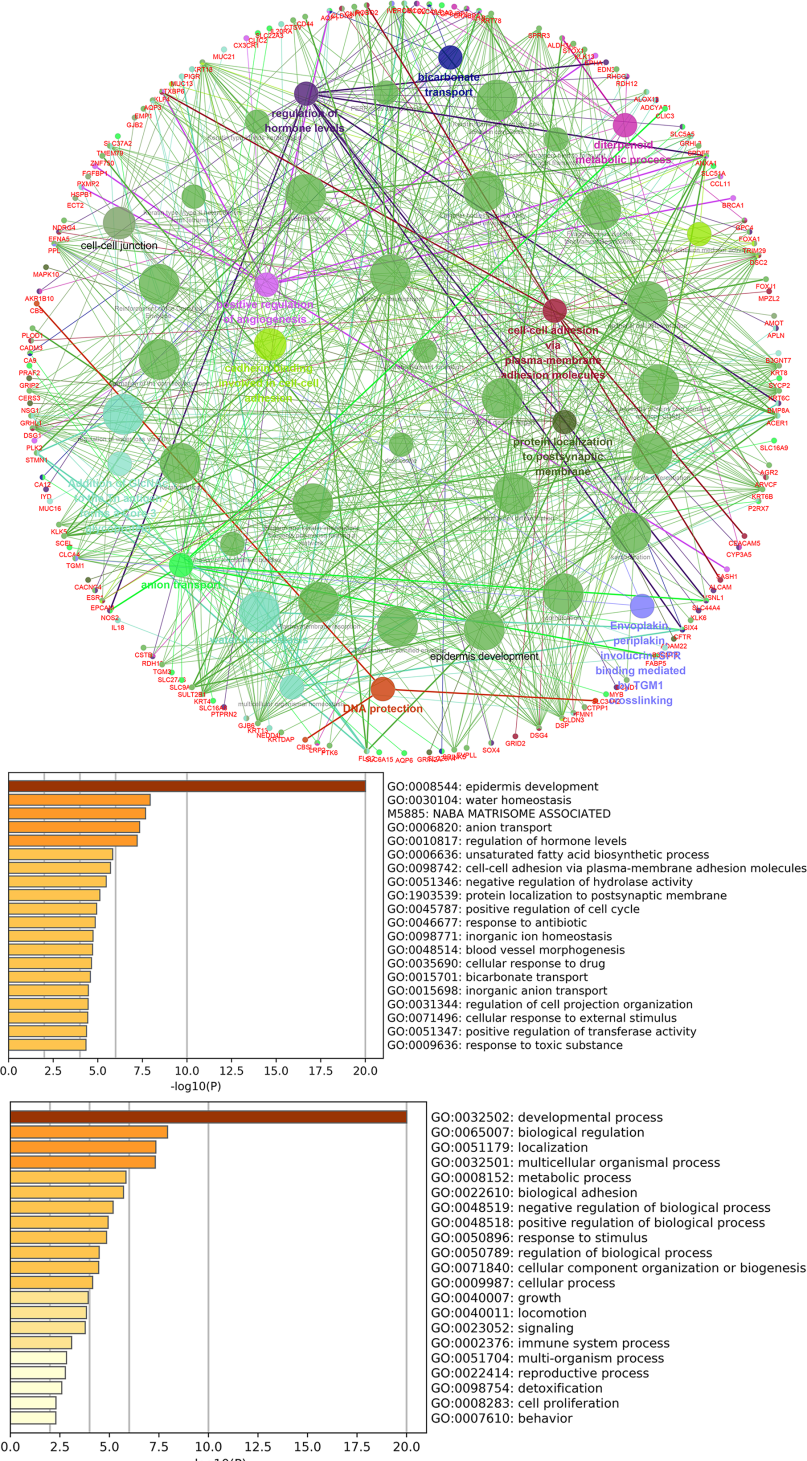
GO and pathway analysis for differentially expressed mRNAs in the highly active subnetwork module.

lncRNAs can regulate the transcription, translation, and splicing of downstream target mRNAs. To understand the correlation between the expression of differentially expressed lncRNAs and differentially expressed mRNAs, a lncRNA-RNA interaction network was constructed. The lncRNA-mRNA coexpression pairs in the network were selected with a threshold of correlation ≥0.95, resulting in a network consisting of 514 nodes and 7,102 significant coexpression relationships, including 127 differentially expressed lncRNAs and 387 differentially expressed mRNAs (**Figure 2A**), suggesting that these differentially expressed lncRNAs might regulate downstream target mRNAs mainly through induction mechanisms. As shown in **Figure 2A**, the coexpression regulatory network was divided into two parts (cis and trans), which are the two regulatory mechanisms by which lncRNAs regulate downstream genes. Then, using the jActive module, we identified a highly active subnetwork module (ActivePath Score = 7.20; **Figure 2B**) from the network, including 66 nodes and 192 edges with 19 lncRNAs and 47 mRNAs. GO and KEGG enrichment analyses were performed to analyze the functions of the differentially expressed mRNAs in each subnetwork module of the network. The results showed that these differentially expressed mRNAs were significantly enriched in epidermal development, regulation of hormone levels, cell cycle, epidermal cell differentiation, phosphorylation, and cell resistance. The main pathways were the HNF3A pathway and regulation of the intracellular estrogen receptor signaling pathway (**Figure 3**), which indicated that these differentially expressed mRNAs may be related to EMT. EMT causes dissociated epithelial cells to acquire migration and invasive capacities and confers cancer cells with the ability to migrate to distant tissues.

### PCBP1-AS1 Is Associated With Poor Prognosis and Clinical Parameters of Cervical Cancer Patients

**Figure 4:**
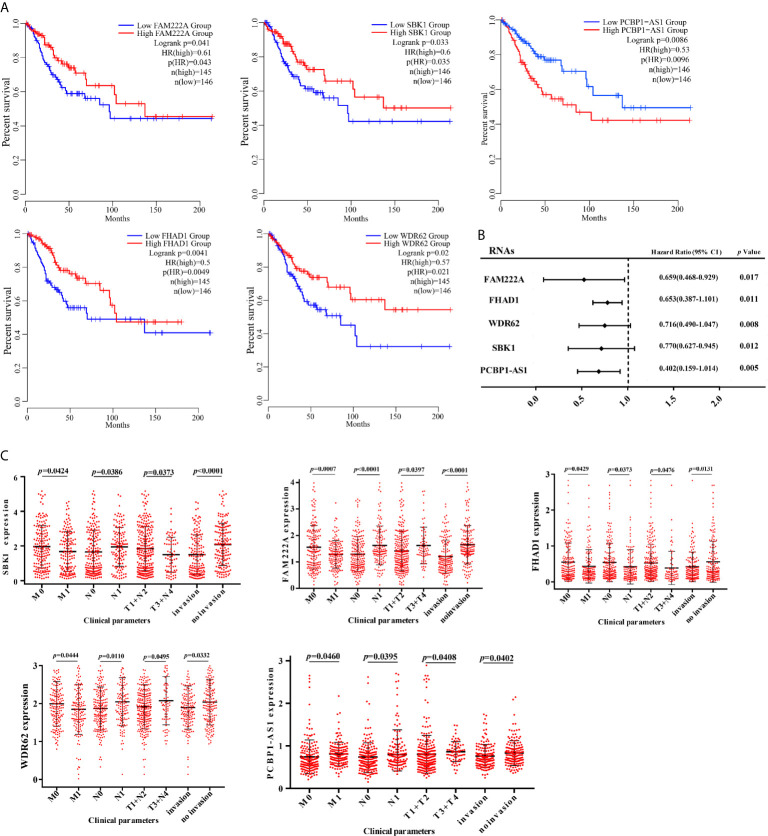
**(A)** Kaplan–Meier analysis results of PCBP1-AS1, FAM222A, FHAD1, WDR62, and SBK1 in CESC. **(B)** Univariate Cox proportional hazards regression analysis of PCBP1-AS1, FAM222A, FHAD1, WDR62, and SBK1 in CESC. **(C)** Expression of PCBP1-AS1, FAM222A, FHAD1, WDR62, and SBK1 correlated significantly with clinicopathological parameters.

To identify the differentially expressed lncRNAs and mRNAs with potential prognostic value, the expression levels of 19 differentially expressed lncRNAs and 47 differentially expressed mRNAs in the network of the subnetwork module were analyzed using a univariate Cox proportional hazards regression model. Only one lncRNA (PCBP1-AS1) and four mRNAs (FAM222A, FHAD1, WDR62, and SBK1) were identified as prognostic factors (*p* < 0.05; **Figure 4B**). Kaplan–Meier curve analysis showed that PCBP1-AS1 was negatively correlated with OS (*p* < 0.05), and all mRNAs were positively correlated with OS (*p* < 0.05) (**Figure 4A**). Meanwhile, we evaluated the relationship among PCBP1-AS1 and four mRNA expression levels and various clinicopathological parameters of cervical cancer patients. Expression data and clinical characteristics were obtained from TCGA-cervical cancer database. The results showed that the expression of PCBP1-AS1, FAM222A, FHAD1, WDR62, and SBK1 was significantly correlated with tumor clinical stage, pathologic TNM, and lymphatic invasion (p < 0.05) (**Figure 4C**). From the multivariate Cox regression analysis, PCBP1-AS1 (*p* = 0.046; HR = 0.407, 95% CI, 0.156–1.06) and SBK1 (*p* = 0.047; HR = 0.804, 95% CI, 0.641–1.007) were independent prognostic factors (**Table 3**, **Supplementary Figure 1**).

**Table 3:** Multivariate cox regression analysis of RNA signature associated with survival in cervical cancer patients.

	Coefficient	HR	SE	P-value
FAM222A	−0.033	0.79	0.197	0.23
FHAD1	−0.029	0.799	0.277	0.418
WDR62	−0.002	0.728	0.2	0.113
SBK1	−0.007	0.804	0.115	0.047
PCBP1-AS1	−0.069	0.407	0.489	0.046

### Expression Validation of PCBP1-AS1 in Cervical Cancer Tissues

**Figure 5:**
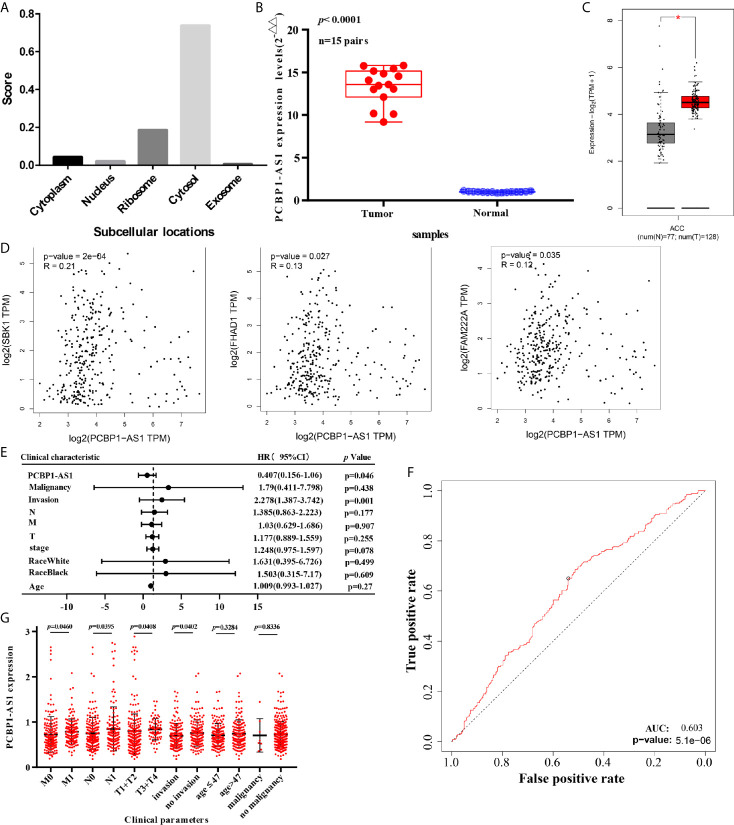
**(A)** Subcellular localizations of PCBP1-AS1 determined by using lncLocator. **(B)** Expression of PCBP1-AS1 in CESC cancer tissues assessed by qPCR. **(C)** Expression of PCBP1-AS1 in CESC from TCGA by GEPIA.*p<0.05. **(D)** Correlation analysis among PCBP1-AS1, FAM222A, FHAD1, and SBK1. **(E)** Multivariate Cox analysis of PCBP1-AS1 expression and other clinicopathological variables. **(F)** ROC curves of PCBP1-AS1. **(G)** Expression of PCBP1-AS1 correlated significantly with clinicopathological parameters.

This project focuses on the regulation of lncRNAs; thus, PCBP1-AS1 was selected as a candidate for further determination of its role in cervical cancer pathogenesis. LncLocator prediction results revealed that PCBP1-AS1 was localized to the cytosol (**Figure 5A**). qRT-PCR was performed to detect PCBP1-AS1 expression levels in 15 cervical cancer tissues and 15 controls. The clinical characteristics of the cervical cancer samples are summarized in **Supplementary Table 1**. As shown in **Figure 5B**, PCBP1-AS1 had significantly higher expression levels in cervical cancer tissues than in their normal counterparts (*p* < 0.001). This result was consistent with the microarray analysis and TCGA data (**Figure 5C**). In addition, the expression of PCBP1-AS1 was positively correlated with FAM222A (*p* = 0.035), FHAD1 (*p* = 0.027), and SBK1 (*p* < 0.001) (**Figure 5D**). Moreover, univariate analysis revealed that PCBP1-AS1 expression, tumor stage, pathologic T stage, and lymph vascular invasion were significantly correlated with the OS of cervical cancer patients (**Table 4-a**). Our multivariate analysis revealed that PCBP1-AS1 expression might be an independent factor for the prognosis of cervical cancer (**Table 4-b**, **Figure 5E**). Meanwhile, the ROC curve AUC of PCBP1-AS1 expression for predicting survival was 0.603 (**Figure 5F**), which indicated that PCBP1-AS1 possessed the potential prognostic ability of cervical cancer. Furthermore, as shown in **Figure 5G**, we uncovered a correlation between PCBP1-AS1 expression and clinicopathologic characteristics. Increased PCBP1-AS1 expression levels in cervical cancer were significantly correlated with tumor stage, pathologic TNM (*p* < 0.05), and lymph invasion (p = 0.0402). These results indicated that cervical cancer patients with high levels of PCBP1-AS1 expression are more likely to promote the initiation and growth of cervical cancer than patients with low levels of PCBP1-AS1 expression due to the effect of tumor stage, pathologic TNM, and lymph invasion.

**Table 4:** Correlation between overall survival and multivariable characteristics *via* (a) univariate analysis (b) multivariate survival analysis.

Clinical characteristic	HR	LOWER	UPER	P
**a**				
Age	1.015	0.997	1.032	0.097
Race Black	1.376	0.289	6.552	0.689
Race White	1.564	0.379	6.447	0.536
Stage	1.494	1.216	1.837	<0.001
T	1.375	1.117	1.694	0.003
M	1.261	0.812	1.958	0.302
N	1.406	0.907	2.181	0.128
Invasion	2.360	1.484	3.753	<0.001
malignancy	1.679	0.408	6.906	0.473
PCBP1-AS1	0.402	0.159	1.014	0.033
**Clinical characteristic**	**HR**	**LOWER**	**UPER**	**P**
**b**				
Age	1.009	0.993	1.027	0.27
Race Black	1.503	0.315	7.17	0.609
Race White	1.631	0.395	6.726	0.499
Stage	1.248	0.975	1.597	0.078
T	1.177	0.889	1.559	0.255
M	1.03	0.629	1.686	0.907
N	1.385	0.863	2.223	0.177
Invasion	2.278	1.387	3.742	0.001
malignancy	1.79	0.411	7.798	0.438
PCBP1-AS1	0.407	0.156	1.06	0.046

### Relationship Between PCBP1-AS1 Expression and Tumor-Infiltrating Immune Cells

**Figure 6:**
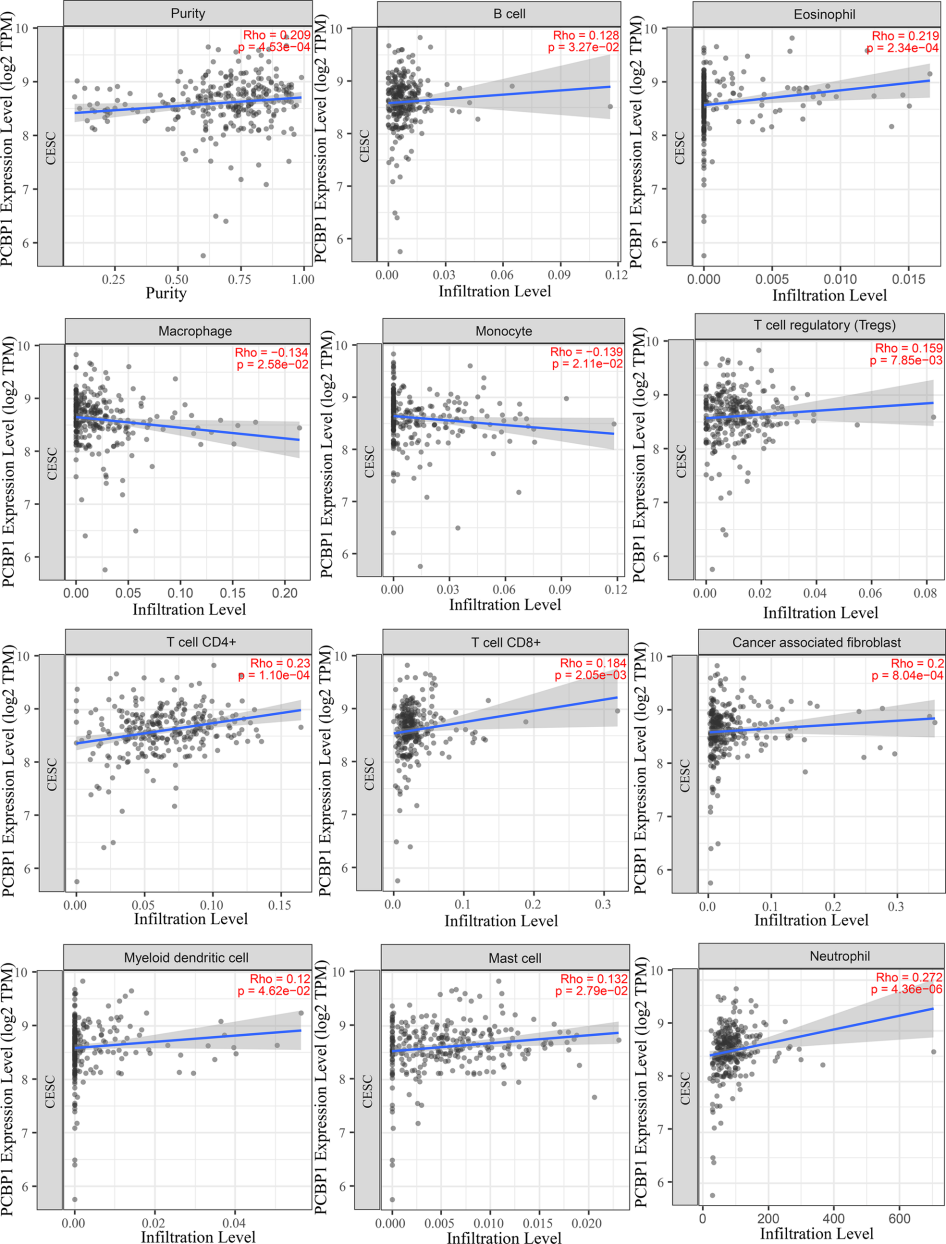
Correlations between PCBP1-AS1 expression and immune infiltration levels, with purity adjustment.

**Figure 7:**
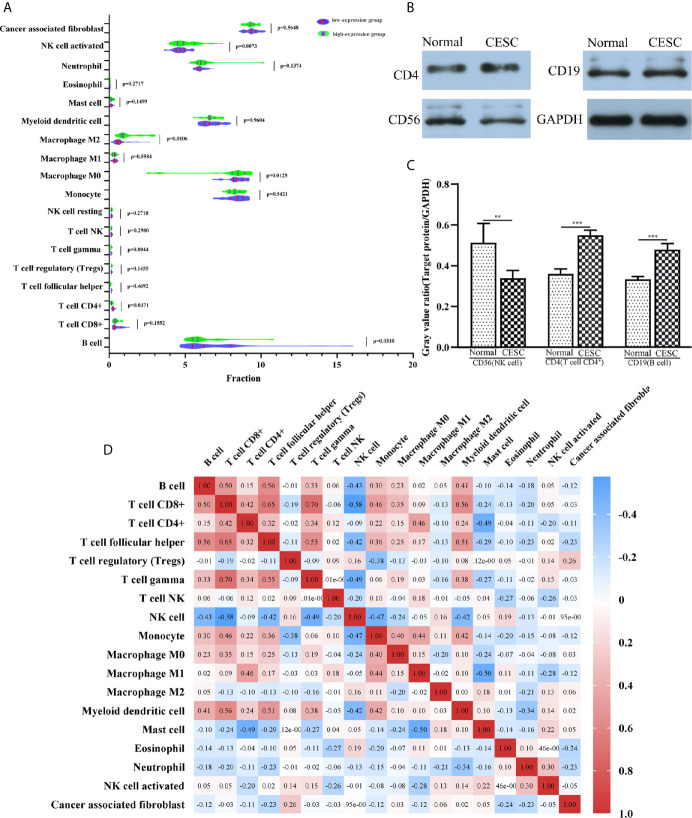
**(A)** The varied proportions of 18 subtypes of immune cells in high and low PCBP1-AS1 expression groups in tumor samples. **(B)** Western blot results of CD4 (representing CD4+ T cells), CD19 (representing B cells), and CD56 (representing NK cells) protein expression in cervical cancer tissues and corresponding adjacent normal cervical cancer tissues. GAPDH was used as a control. **(C)** Gray value ratios of CD4/GAPDH, CD19/GAPDH, and CD56/GAPDH in cervical cancer tissues and corresponding adjacent normal cervical cancer tissues. **p < 0.01,***p < 0.001. **(D)** Heatmap of 18 infiltrating immune cells in tumor samples.

To understand the influence of PCBP1-AS1 in tumor-infiltrating lymphocytes, we analyzed the possible correlations between PCBP1-AS1 expression and levels of immune infiltration in cervical cancer. As shown in **Figure 6**, PCBP1-AS1 expression showed a positive correlation with the levels of CD8^+^ T cells (*p* < 0.05), CD4^+^ T cells (*p* < 0.05), B cells (*p* < 0.05), cancer-associated fibroblasts (*p* < 0.05), myeloid dendritic cells (*p* < 0.05), eosinophils (*p* < 0.05), mast cells (*p* < 0.05), neutrophils (*p* < 0.05), and regulatory T cells (*p* < 0.05). In contrast, the presence of macrophages and monocytes was negatively correlated with the levels of PCBP1-AS1 expression. The results indicated that PCBP1-AS1 played an important role in immune infiltration in cervical cancer. Meanwhile, to study whether the cervical cancer immune microenvironment was different in cases with high PCBP1-AS1 levels compared those with to low levels, we downloaded an RNA expression profile obtained from TCGA. The cervical cancer samples were divided into two groups with the median value of PCBP1-AS1 expression as a cutoff. Then, we explored the expression profiles to obtain a fraction of 18 immune cell subtypes and assessed the differences in their expression levels in the two PCBP1-AS1 expression groups (**Figure 7A**). B cells, CD4+ T cells, M0 macrophages, M2 macrophages, and activated NK cells were significantly affected by PCBP1-AS1 expression. M2 macrophages and activated NK cells were increased (p < 0.05) in the low expression group compared to the high expression group. In contrast, CD4+ T cells, B cells, and M0 macrophages were increased in the high expression group (p < 0.05). We collected five pairs of samples (five cervical cancer tissues and five adjacent tissues) and extracted total protein from them. Differences in CD4+ T cells (CD4), B cells (CD19), and NK cells (CD56) in normal samples and cervical cancer samples were detected by western blotting. The results showed that CD56 protein was present at low levels in the tumor samples. In contrast, CD4 and CD19 protein levels were higher in tumor samples than in normal samples, which indirectly confirmed the results of the above analysis of immune infiltration (**Figures 7B, C**). In addition, we analyzed the correlations between 18 types of immune cells (**Figure 7D**), which revealed that the different infiltrating immune cell subpopulations of cervical cancer were moderately correlated.

### Gene Set Enrichment Analysis of PCBP1-AS1

**Figure 8:**
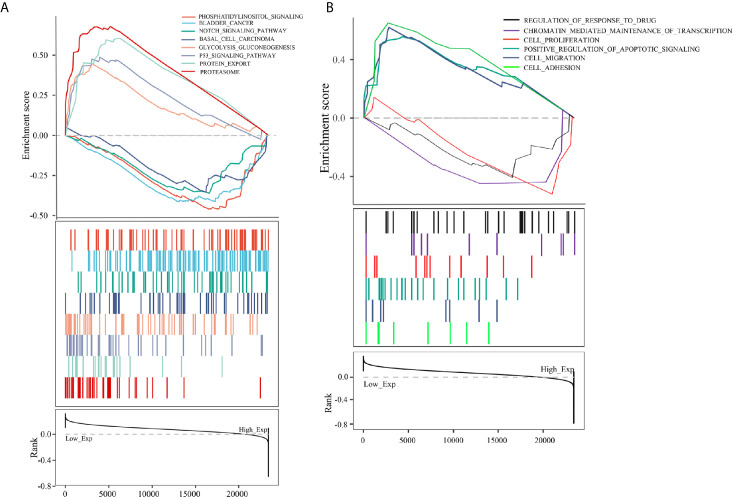
**(A)** KEGG pathway analysis showed five positively correlated groups and five negatively correlated groups. **(B)** GO term analysis revealed three positively correlated groups and three negatively correlated groups.

To further analyze the function of PCBP1-AS1, GSEA was performed, and the most differentially (FDR q-val < 0.250, NOM p-val < 0.050) enriched signaling pathways and functions were selected based on the normalized enrichment score (NES). As shown in **Figure 8B**, the GO sets of molecular functions and biological processes significantly associated with PCBP1-AS1 expression were cell adhesion, cell migration, cell proliferation, regulation of apoptosis, cell resistance, and chromatin regulation. KEGG pathway analysis showed that the four pathways with the strongest positive correlations with PCBP1-AS1 expression were protein export, proteasome, p53 signaling pathway, and glycolysis gluconeogenesis; the four pathways with the strongest negative correlations were phosphatidylinositol signaling, basal cell carcinoma, bladder cancer, and Notch signaling, as shown in **Figure 8A**. The above GO and KEGG pathway annotations are shown in **Table 5**. These results revealed that the expression level of PCBP1-AS1 was strongly associated with GO functions and pathways regulating cell function (cell adhesion, migration, proliferation, apoptosis, and resistance) and chromosome and protein activity.

**Table 5:** Signaling pathways most significantly correlated with PCBP1-AS1 expression based on their normalized enrichment score (NES) and p-value.

	GO name	NES	NOM p-value
Positive	Cell adhesion	1.60	0.04
	Cell migration	1.59	0.027
	Negative regulation of apoptotic	1.62	0.032
Negative	Positive regulation of response to drug	−1.53	0.037
	Chromatin mediated maintenance of transcription	−1.61	0.024
	Cell proliferation	−1.58	0.032
	**KEGG name**	**NES**	**NOM p-value**
Positive	Protein export	1.63	0.046
	Proteasome	1.70	0.034
	p53 signaling pathway	1.52	0.032
	Glycolysis gluconeogenesis	1.57	0.035
Negative	Phosphatidylinositol signaling	−1.90	<0.001
	Basal cell carcinoma	−1.37	0.011
	Bladder cancer	−1.71	0.010
	Notch signaling	−1.43	0.044

### PCBP1-AS1 Contributes to HeLa Cell Proliferation, Migration, and Invasion

**Figure 9:**
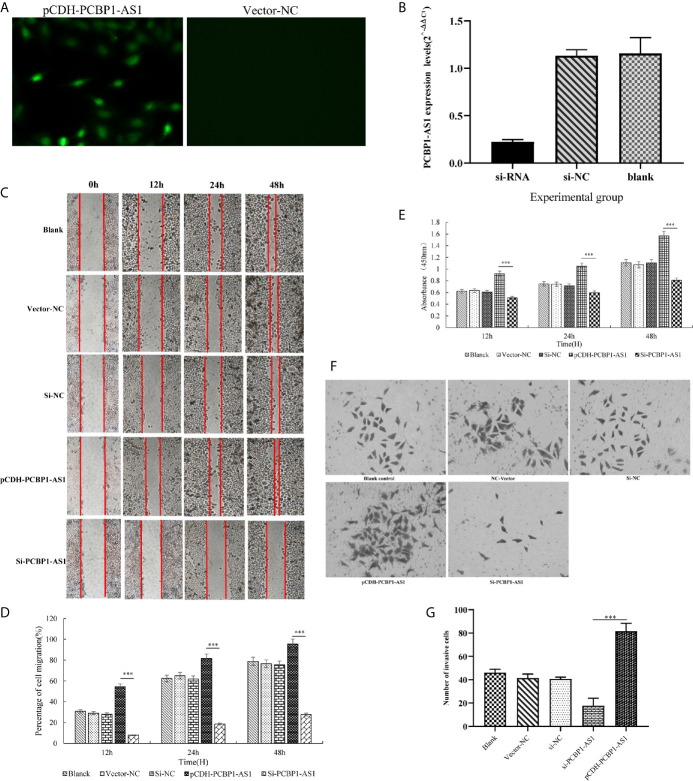
**(A)** Overexpression of PCBP1-AS1 in HeLa cells analyzed by fluorescence microscopy. **(B)** PCBP1-AS1 knockout efficiency in HeLa cells analyzed by qPCR. **(C)** and **(D)** The effects of PCBP1-AS1 knockdown or overexpression on HeLa cell migration measured using the migration assay. ^⁎⁎⁎^ *p* < 0.001. **(E)** The effects of PCBP1-AS1 knockdown and overexpression on the viability of HeLa cells measured using the CCK-8 assay. **(F)** and **(G)** Transwell invasion assay and average number of invasive PCBP1-AS1 knockdown and overexpression cells. ^⁎⁎⁎^p < 0.001.

To further address the biological function of PCBP1-AS1, we used siRNA and overexpression plasmids to alter the expression level of PCBP1-AS1 in HeLa cells and analyzed the effect of PCBP1-AS1 on HeLa cell proliferation, migration, and invasion. Fluorescence microscopy analysis of PCBP1-AS1 revealed successful transfection of the overexpression plasmid (**Figure 9A**), and siRNA‐PCBP1-AS1 led to a significant decrease in PCBP1-AS1 expression in HeLa cells (**Figure 9B**). The effect of PCBP1-AS1 on cell proliferation was detected by the CCK-8 assay. Compared with the negative control (NC) group, overexpression of PCBP1-AS1 significantly promoted HeLa cell proliferation, whereas HeLa cell proliferation was significantly impaired by PCBP1-AS1 knockdown (*p* < 0.001) (**Figure 9E**). Meanwhile, the wound-healing assay revealed that overexpression of PCBP1-AS1 significantly enhanced HeLa cell migration, and siRNA-PCBP1-AS1 showed a notably slower scratch closure rate than control cells (**Figures 9C, D**), which revealed that silencing PCBP1-AS1 inhibited HeLa cell migration (*p* < 0.001). Furthermore, the Transwell assay demonstrated that PCBP1-AS1 knockdown HeLa cells displayed significantly lower invasion potential than the control cells (*p* < 0.001) (**Figures 9F, G**). Collectively, these results suggest that the expression level of PCBP1-AS1 affected the proliferation, migration, and invasion of cervical cancer cells.

## Discussion

Cervical cancer is one of the most common malignancies in females, and it has the highest mortality among female reproductive system malignancies (1, 2). Despite the development of diagnostic and treatment strategies, the prognosis of cervical cancer patients is still very poor, mainly due to cancer metastasis and recurrence (5–7). Thus far, a series of studies have indicated that lncRNAs exert substantial effects on the pathogenesis of carcinomas, suggesting that lncRNAs might act as prognostic indicators in tumorigenesis and cancer development (14–16). For example, PSMB8-AS1 contributes to pancreatic cancer progression by modulating the miR-382-3p/STAT1/PD-L1 axis (21). However, limited research has been performed on the transcriptomic profiles of cervical cancer, and the functional roles of lncRNAs in cervical cancer pathogenesis remain largely unknown. Hence, comprehensively understanding the lncRNA profile of cervical cancer and analyzing the mechanism of action involving lncRNAs might provide new thinking in the pathogenesis of this disease.

In this study, high-throughput microarray analysis was performed to characterize the significantly differentially expressed lncRNAs and mRNAs between cervical cancer patients and controls, which might be involved in cervical cancer progression. In total, 130 lncRNAs and 656 mRNAs were found to be dysregulated. The functions of lncRNAs are closely associated with downstream target mRNAs, which they may regulate directly or indirectly (22, 23). Therefore, a global lncRNA-mRNA coexpression cis- and trans-regulatory network was constructed with 127 differentially expressed lncRNAs and 387 differentially expressed mRNAs, which could be successfully used for disease-related lncRNA identification. Based on the lncRNA-PCG functional network, we identified a highly active subnetwork module, including 19 lncRNAs and 47 mRNAs, by the jActive module. GO and KEGG enrichment analysis based on the 47 differentially expressed mRNAs indicated that several biological processes and pathways may play important roles in cervical cancer pathogenesis, including epidermal development, cell cycle, cell resistance, epidermal cell differentiation, and regulation of the intracellular estrogen receptor signaling pathway, which indicated that these DEmRNAs may be related to EMT. EMT causes dissociated epithelial cells to acquire migratory and invasive capacities and endows cancer cells with the ability to migrate to distant tissues. This functional annotation provides bioinformatics-based evidence regarding the potential mechanism promoting cervical cancer occurrence. Based on Kaplan‐Meier analysis, PCBP1-AS1 and four mRNAs (FAM222A, FHAD1, WDR62, and SBK1) were identified as potential prognostic factors for cervical cancer patients. The expression of these DERNAs was significantly correlated with tumor clinical stage, pathologic TNM, and lymphatic invasion.

To the best of our knowledge, the five DERNAs have rarely been reported in previous studies, and their functions in cervical cancer are largely unknown. Originally, PCBP1-AS1 was identified in cervical cancer tissues through microarray expression profiling (24). However, its expression and biological function in cervical cancer tissues and cells have not been studied. Luo et al. reported that PCBP1-AS1 aggravated the progression of hepatocellular carcinoma by regulating the PCBP1/PRL-3/AKT pathway (25). Luan et al. noted that PCBP1-AS1 promoted the autophagy of glioma cells (26). FAM222A is Chromosome 12 Open Reading Frame 34. It was reported that FAM222a is related to chemotherapy resistance in gastric cancer, and its antisense RNA can regulate the migration of non-small cell lung cancer cells (27, 28). FHAD1 was reported by Zhao et al. to be a marker for the occurrence of prostate cancer (29). WDR62 was identified as a scaffold protein in the JNK signaling pathway (30). Zhou found that inactivation of WDR62 could cause defects in female meiotic initiation, which led to the occurrence of female reproductive diseases (31). SBK1 is also a peptide domain. Wang found that SBK1 was dysregulated in several cancer tissues, especially in ovarian cancer, and showed that SBK1 played an important role during ovarian carcinogenesis (32). According to the above studies, these five hub genes are likely to be involved in the occurrence or development of cervical cancer.

Our research results showed that PCBP1-AS1 expression was increased in cervical cancer tissues compared with paired adjacent normal tissues and was a prognostic biomarker for cervical cancer. Additionally, we found a role for upregulated PCBP1-AS1 as an independent prognostic factor for poor OS. Cervical cancer patients with high PCBP1-AS1 expression are more likely to have a more advanced stage, TNM status, and lymph metastasis than those with low PCBP1-AS1 expression. In addition, we analyzed the connections between PCBP1-AS1 expression and immune infiltration levels in cervical cancer by TIMER. We found a relationship between PCBP1-AS1 and T cell, B cell, myeloid dendritic cell, eosinophil, mast cell, neutrophil, macrophage, and monocyte infiltration. Furthermore, the immune infiltration score analysis showed that B cells, CD4+ T cells, M0 macrophages, M2 macrophages, and NK cells were related to PCBP1-AS1 expression. The results revealed that CD4+, B cells, and M0 macrophages were increased in the high expression group, whereas the levels of M2 macrophages and activated NK cells were decreased. It is reported that NK cells are important biological barriers that are resident in the cervix and can identify and kill virus-infected cells rapidly through pathways that do not require preactivation (33). Other research suggests that HPV16 disables the increased NK cells in the early lesion of the cervix (34). Here, we speculate that these phenomena may be a possible mechanism by which PCBP1-AS1 regulates the functions of NK cells in cervical cancer. Furthermore, the overexpression of PCBP1-AS1 may inhibit efficient NK cell immune responses and infiltration. Overall, PCBP1-AS1 plays a crucial role in the regulation and recruitment of immune infiltrating cells in cervical cancer. However, these results need to be further validated in combination with clinical trials.

Equally important, we performed GSEA to further analyze the biological function of PCBP1-AS1. Our results showed that the main significant pathways for PCBP1-AS1 included the p53 signaling pathway and Notch signaling. Notch signaling is well known to be one of the most frequently activated signaling pathways in cancer and is involved in cell cycle regulation (35) and immune responses (36). Rong et al. reported that activated Notch signaling may lead to the development of cervical cancer by regulating Numb splicing (37). Similarly, P53 also plays a complex role in promoting the cell cycle, cell senescence, and apoptosis. Wild-type p53 can promote the cancer metabolic switch by inducing PUMA-dependent suppression of oxidative phosphorylation (38). Furthermore, recent research found that many more genes could promote proliferation and suppress apoptosis in cervical cancer cells by inhibiting and activating the p53 signaling pathway (39–41). Our results help to deepen the understanding of the biological functions of PCBP1-AS1 in cervical cancer. Furthermore, to validate the identified biological functions, gain-of-function experiments were performed in HeLa cells. Our results showed that PCBP1-AS1 depletion could significantly inhibit cervical cancer cell proliferation. Wound healing assays and Transwell assays further demonstrated that downregulation of PCBP1-AS1 could reduce the migration and invasion ability of cervical cancer cells. Collectively, these results provide mechanistic evidence supporting the finding that PCBP1-AS1 upregulation is associated with more advanced stage, TNM status, and lymph metastasis. PCBP1-AS1 may also be a useful biomarker for cervical cancer.

In conclusion, we used transcriptome sequencing technology to profile the lncRNAs of both cervical cancer and adjacent mucosa from 15 patients. A total of 130 lncRNAs and 656 mRNAs were systematically screened, many of which played important roles in regulating cell biological functions. These sequencing data provide an important resource for future studies of key lncRNAs in cervical cancer. Of these, PCBP1-AS1 was found to be a new biomarker for the prognosis of cervical cancer patients and to regulate cell proliferation and migration. In addition, this study helps to elucidate the roles of immune cell infiltration and lncRNAs in cervical cancer. With a better understanding of the biological function of PCBP1-AS1, this molecule could act as an effective biomarker for the diagnosis and treatment of cervical cancer and may help clinicians make appropriate choices for targeted therapy for the treatment of cervical cancer in the future.

## Data Availability Statement

The raw sequencing dates presented in the study are publicly available, which has been uploaded to Gene Expression Omnibus (GEO). These data can be found here: https://www.ncbi.nlm.nih.gov/geo/query/acc.cgi?acc=GSE167362.

## Ethics Statement

The studies involving human participants were reviewed and approved by the Ethics Committee of Jiangning Hospital of Nanjing Medical University. The patients/participants provided their written informed consent to participate in this study.

## Author Contributions

QL and LHL were the principal investigators who designed and conceived the study and obtained financial support. LHL and QP analyzed the data and wrote the manuscript. YX, LL, and MG prepared the dataset. All authors contributed to the article and approved the submitted version.

## Conflict of Interest

The authors declare that the research was conducted in the absence of any commercial or financial relationships that could be construed as a potential conflict of interest.

## Supplementary Material

Supplementary Figure 1 Prognostic risk score model analysis of PCBP1-AS1 and SBK1 in CESC patients. **(A)** From top to bottom: risk score distribution, patient survival status distribution, and heatmap of PCBP1-AS1 and SBK1 expression profiles ranked by risk score. **(B)** Kaplan–Meier curves for high-risk and low-risk groups. **(C)** The ROC curves for predicting survival in CESC patients by the risk score.

## References

[1] Shafabakhsh R Reiter RJ Mirzaei H Teymoordash SN Asemi Z. Melatonin: A new inhibitor agent for cervical cancer treatment. J Cell Physiol (2019) 234:21670–82. 10.1002/jcp.28865 31131897

[2] Vu M Yu J Awolude OA Chuang L. Cervical cancer worldwide. Curr Probl Cancer (2018) 42:457–65. 10.1016/j.currproblcancer.2018.06.003 30064936

[3] Wei M Zhou W Bi Y Wang H Liu Y Zhang ZJ. Rising Mortality Rate of Cervical Cancer in Younger Women in Urban China. J Gen Intern Med (2019) 34:281–4. 10.1007/s11606-018-4732-z PMC6374275 30484099

[4] Small W Bacon MA Bajaj A Chuang LT Fisher BJ Harkenrider MM. Cervical cancer: A global health crisis. Cancer (2017) 123:2404–12. 10.1002/cncr.30667 28464289

[5] Koh WJ Abu-Rustum NR Bean S Bradley K Campos SM Cho KR. Cervical Cancer, Version 3.2019, NCCN Clinical Practice Guidelines in Oncology. J Natl Compr Canc Netw (2019) 17:64–84. 10.6004/jnccn.2019.0001 30659131

[6] Mallmann P Mallmann C. Neoadjuvant and Adjuvant Chemotherapy of Cervical Cancer. Oncol Res Treat (2016) 39:522–4. 10.1159/000449023 27614740

[7] Vordermark D. Radiotherapy of Cervical Cancer. Oncol Res Treat (2016) 39:516–20. 10.1159/000448902 27614991

[8] Cancer Genome Atlas Research Network. Albert Einstein College of Medicine; Analytical Biological Services; Integrated genomic and molecular characterization of cervical cancer. Nature (2017) 543:378–84. 10.1038/nature21386 PMC5354998 28112728

[9] Minion LE Tewari KS. Cervical cancer - State of the science: From angiogenesis blockade to checkpoint inhibition. Gynecol Oncol (2018) 148:609–21. 10.1016/j.ygyno.2018.01.009 PMC6720107 29666026

[10] ENCODE Project Consortium Birney E Stamatoyannopoulos JA. Identification and analysis of functional elements in 1% of the human genome by the ENCODE pilot project. Nature (2007) 447:799–816. 10.1038/nature05874 17571346 PMC2212820

[11] Outeiro-Pinho G Barros-Silva D Correia MP Henrique R Jerónimo C. Renal Cell Tumors: Uncovering the Biomarker Potential of ncRNAs. Cancers (Basel) (2020) 12(8):2214. 10.3390/cancers12082214 PMC7465320 32784737

[12] Yi J Li S Wang C Cao N Qu H Cheng C. Potential applications of polyphenols on main ncRNAs regulations as novel therapeutic strategy for cancer. BioMed Pharmacother (2019) 113:108703. 10.1016/j.biopha.2019.108703 30870719

[13] Feng W Su Z Yin Q Zong W Shen X Ju S. ncRNAs associated with drug resistance and the therapy of digestive system neoplasms. J Cell Physiol (2019) 234:19143–57. 10.1002/jcp.28551 30941775

[14] Hu Q Ye Y Chan LC Li Y Liang K Lin A. Oncogenic lncRNA downregulates cancer cell antigen presentation and intrinsic tumor suppression. Nat Immunol (2019) 20:835–51. 10.1038/s41590-019-0400-7 PMC6619502 31160797

[15] Dong P Xiong Y Yue J B Hanley S Kobayashi N Todo Y. Exploring lncRNA-Mediated Regulatory Networks in Endometrial Cancer Cells and the Tumor Microenvironment: Advances and Challenges. Cancers (Basel) (2019) 11:234. 10.3390/cancers11020234 PMC6406952 30781521

[16] Jiang MC Ni JJ Cui WY Wang BY Zhuo W. Emerging roles of lncRNA in cancer and therapeutic opportunities. Am J Cancer Res (2019) 9:1354–66. PMC6682721 31392074

[17] Liu Y Zhang R Qiu F Li K Zhou Y Shang D. Construction of a lncRNA-PCG bipartite network and identification of cancer-related lncRNAs: a case study in prostate cancer. Mol Biosyst (2015) 11:384–93. 10.1039/c4mb00439f 25385343

[18] Wang P Ning S Zhang Y Li R Ye J Zhao Z. Identification of lncRNA-associated competing triplets reveals global patterns and prognostic markers for cancer. Nucleic Acids Res (2015) 43:3478–89. 10.1093/nar/gkv233 PMC4402541 25800746

[19] Wang J Li Z Gao A Wen Q Sun Y. The prognostic landscape of tumor-infiltrating immune cells in cervical cancer. BioMed Pharmacother (2019) 120:109444. 10.1016/j.biopha.2019.109444 31562978

[20] Luo W Wang M Liu J Cui X Wang H. Identification of a six lncRNAs signature as novel diagnostic biomarkers for cervical cancer. J Cell Physiol (2020) 235:993–1000. 10.1002/jcp.29015 31332778

[21] Zhang H Zhu C He Z Chen S Li L Sun C. LncRNA PSMB8-AS1 contributes to pancreatic cancer progression via modulating miR-382-3p/STAT1/PD-L1 axis. J Exp Clin Cancer Res (2020) 39:179. 10.1186/s13046-020-01687-8 32891166 PMC7487636

[22] Zhang Y Xu Y Feng L Li F Sun Z Wu T. Comprehensive characterization of lncRNA-mRNA related ceRNA network across 12 major cancers. Oncotarget (2016) 7:64148–67. 10.18632/oncotarget.11637 PMC5325432 27580177

[23] Liu XD Xie DF Wang YL Guan H Huang RX Zhou PK. Integrated analysis of lncRNA-mRNA co-expression networks in the α-particle induced carcinogenesis of human branchial epithelial cells. Int J Radiat Biol (2019) 95:144–55. 10.1080/09553002.2019.1539880 30395764

[24] Campos-Parra AD Padua-Bracho A Pedroza-Torres A Figueroa-González G Fernández-Retana J Millan-Catalan O. Comprehensive transcriptome analysis identifies pathways with therapeutic potential in locally advanced cervical cancer. Gynecol Oncol (2016) 143:406–13. 10.1016/j.ygyno.2016.08.327 27581326

[25] Luo T Gao Y Zhangyuan G Xu X Xue C Jin L. lncRNA PCBP1-AS1 Aggravates the Progression of Hepatocellular Carcinoma via Regulating PCBP1/PRL-3/AKT Pathway. Cancer Manag Res (2020) 12:5395–408. 10.2147/CMAR.S249657 PMC7352448 32753957

[26] Luan F Chen W Chen M Yan J Chen H Yu H. An autophagy-related long non-coding RNA signature for glioma. FEBS Open Bio (2019) 9:653–67. 10.1002/2211-5463.12601 PMC6443865 30984540

[27] Zhang X Han J Du L Li X Hao J Wang L. Unique metastasis-associated lncRNA signature optimizes prediction of tumor relapse in lung adenocarcinoma. Thorac Cancer (2020) 11(3):728–37. 10.1111/1759-7714.13325 PMC7049496 31994347

[28] Choi SJ Jung SW Huh S Chung YS Cho H Kang H. Alteration of DNA Methylation in Gastric Cancer with Chemotherapy. J Microbiol Biotechnol (2017) 27(8):1367–78. 10.4014/jmb.1704.04035 28621113

[29] Zhao S Geybels MS Leonardson A Rubicz R Kolb S Yan Q. Epigenome-Wide Tumor DNA Methylation Profiling Identifies Novel Prognostic Biomarkers of Metastatic-Lethal Progression in Men Diagnosed with Clinically Localized Prostate Cancer. Clin Cancer Res (2017) 23(1):311–9. 10.1158/1078-0432.CCR-16-0549 PMC5199634 27358489

[30] Wasserman T Katsenelson K Daniliuc S Hasin T Choder M Aronheim A. A novel c-Jun N-terminal kinase (JNK)-binding protein WDR62 is recruited to stress granules and mediates a nonclassical JNK activation. Mol Biol Cell (2010) 21(1):117–30 e09–06-0512. 10.1091/mbc PMC2801705 19910486

[31] Zhou Y Qin Y Qin Y Xu B Guo T Ke H. Wdr62 is involved in female meiotic initiation via activating JNK signaling and associated with POI in humans. PloS Genet (2018) 14(8):e1007463. 10.1371/journal.pgen.1007463 30102701 PMC6107287

[32] Wang P Guo J Wang F Shi T Ma D. Human SBK1 is dysregulated in multiple cancers and promotes survival of ovary cancer SK-OV-3 cells. Mol Biol Rep (2011) 38(5):3551–9. 10.1007/s11033-010-0465-8 21104019

[33] Renoux VM Bisig B Langers I Dortu E Clémenceau B Thiry M. Human papillomavirus entry into NK cells requires CD16 expression and triggers cytotoxic activity and cytokine secretion. Eur J Immunol (2011) 41:3240–52. 10.1002/eji.201141693 21830210

[34] Zhang J Jin S Li X Liu L Xi L Wang F. Human Papillomavirus Type 16 Disables the Increased Natural Killer Cells in Early Lesions of the Cervix. J Immunol Res (2019) 2019:9182979. 10.1155/2019/9182979 31183395 PMC6512046

[35] Qin J Wang R Zhao C Wen J Dong H Wang S. Notch signaling regulates osteosarcoma proliferation and migration through Erk phosphorylation. Tissue Cell (2019) 59:51–61. 10.1016/j.tice.2019.07.002 31383289

[36] Vieceli Dalla Sega F Fortini F Aquila G Campo G Vaccarezza M Rizzo P. Notch Signaling Regulates Immune Responses in Atherosclerosis. Front Immunol (2019) 10:1130. 10.3389/fimmu.2019.01130 31191522 PMC6540611

[37] Rong C Feng Y Ye Z. Notch is a critical regulator in cervical cancer by regulating Numb splicing. Oncol Lett (2017) 13:2465–70. 10.3892/ol.2017.5683 PMC5403445 28454421

[38] Kim J Yu L Chen W Xu Y Wu M Todorova D. Wild-Type p53 Promotes Cancer Metabolic Switch by Inducing PUMA-Dependent Suppression of Oxidative Phosphorylation. Cancer Cell (2019) 35:191–203.e8. 10.1016/j.ccell.2018.12.012 30712844

[39] Gao X Li Q Chen G He H Ma Y. MAGEA3 promotes proliferation and suppresses apoptosis in cervical cancer cells by inhibiting the KAP1/p53 signaling pathway. Am J Transl Res (2020) 12:3596–612. PMC7407682 32774721

[40] Liu Y Li L Liu Y Geng P Li G Yang Y. RECK inhibits cervical cancer cell migration and invasion by promoting p53 signaling pathway. J Cell Biochem (2018) 119:3058–66. 10.1002/jcb.26441 29064588

[41] Cui L Nai M Zhang K Li L Li R. lncRNA WT1-AS inhibits the aggressiveness of cervical cancer cell via regulating p53 expression via sponging miR-330-5p. Cancer Manag Res (2019) 11:651–67. 10.2147/CMAR.S176525 PMC6331070 30666161

