# Supplementary figures and images for: Using lncRNA Sequencing to Reveal a Putative lncRNA-mRNA Correlation Network and the Potential Role of PCBP1-AS1 in the Pathogenesis of Cervical Cancer
Source: Front Oncol. 2021 Mar 23;11:634732. doi: 10.3389/fonc.2021.634732 (PMC8023048; doi:10.3389/fonc.2021.634732)

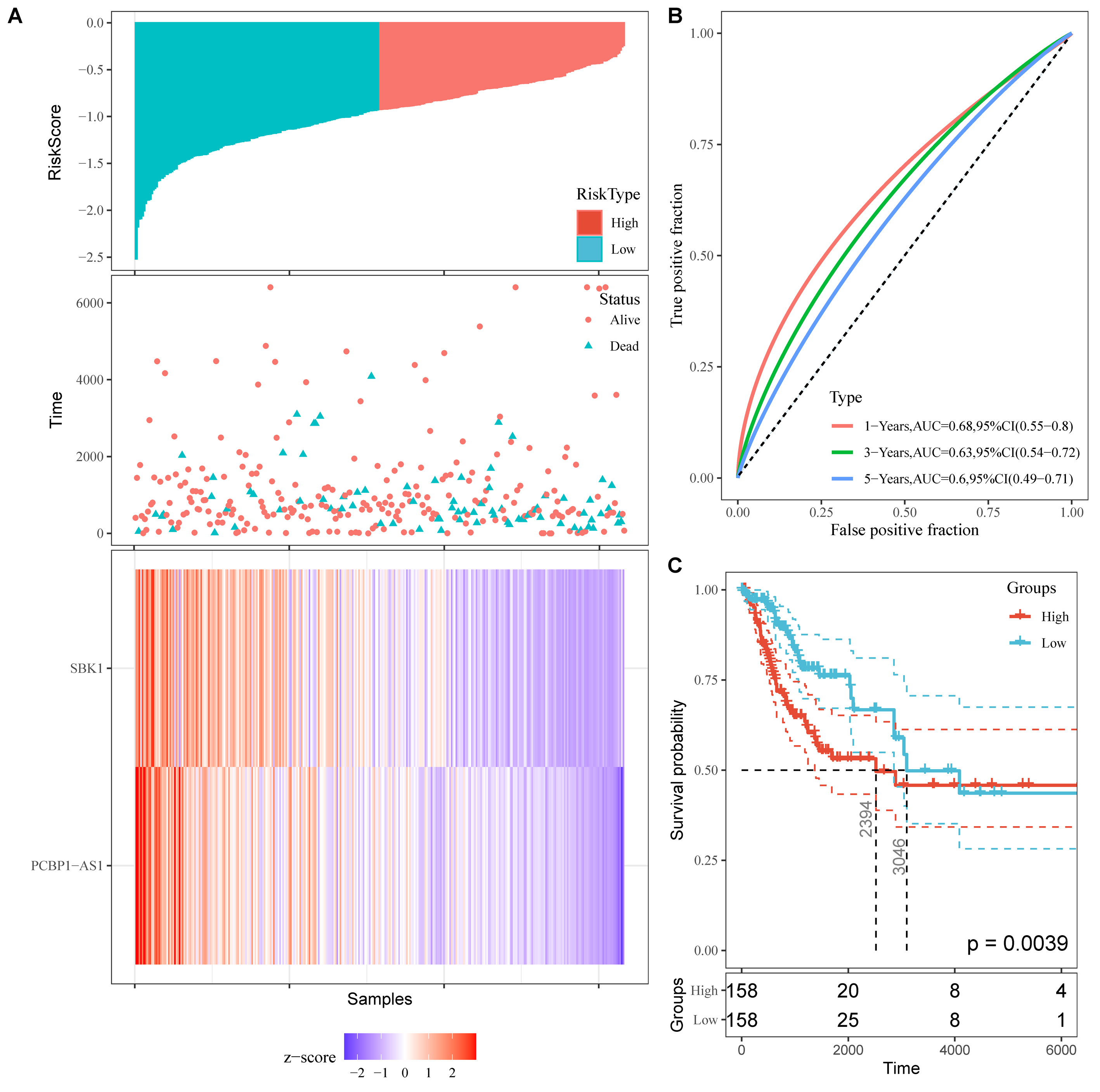

Supplement: Supplementary Figure 1 — Prognostic risk score model analysis of PCBP1-AS1 and SBK1 in CESC patients. (A) From top to bottom: risk score distribution, patient survival status distribution, and heatmap of PCBP1-AS1 and SBK1 expression profiles ranked by risk score. (B) Kaplan–Meier curves for high-risk and low-risk groups. (C) The ROC curves for predicting survival in CESC patients by the risk score. [file Image_1.tif]
