# Supplementary material for: Using lncRNA Sequencing to Reveal a Putative lncRNA-mRNA Correlation Network and the Potential Role of PCBP1-AS1 in the Pathogenesis of Cervical Cancer
Source: Front Oncol. 2021 Mar 23;11:634732. doi: 10.3389/fonc.2021.634732 (PMC8023048; doi:10.3389/fonc.2021.634732)
Supplement: Supplementary file 2 [file Table_1.doc]

Supplementary Table 1. The basic characteristics of 20 cervical cancer samples (adjacent tissues were provided by the same patient).

| Sample Num | Age (years) | Stage | Lymphatic Metastasis (Yes/No) | TNM |
| --- | --- | --- | --- | --- |
| CESC-1 | 56 | II | No | N |
| CESC-2 | 73 | III | No | N |
| CESC-3 | 57 | III | No | M |
| CESC-4 | 71 | I | No | M |
| CESC-5 | 68 | II | No | M |
| CESC-6 | 65 | III | No | T |
| CESC-7 | 46 | I | No | N |
| CESC-8 | 76 | II | No | T |
| CESC-9 | 68 | III | No | M |
| CESC-10 | 76 | III | No | M |
| CESC-11 | 56 | I | No | T |
| CESC-12 | 78 | I | No | N |
| CESC-13 | 52 | II | No | T |
| CESC-14 | 67 | III | No | M |
| CESC-15 | 63 | I | No | N |
| CESC-16 | 60 | III | No | M |
| CESC-17 | 47 | II | No | N |
| CESC-18 | 54 | III | No | M |
| CESC-19 | 53 | III | No | N |
| CESC-20 | 61 | II | No | N |

GC: cervical cancer samples; TNM: tumor-node metastasis.
