# Supplementary material for: Using lncRNA Sequencing to Reveal a Putative lncRNA-mRNA Correlation Network and the Potential Role of PCBP1-AS1 in the Pathogenesis of Cervical Cancer
Source: Front Oncol. 2021 Mar 23;11:634732. doi: 10.3389/fonc.2021.634732 (PMC8023048; doi:10.3389/fonc.2021.634732)
Supplement: Supplementary file 3 [file Table_2.docx]

| **sample** | **Raw reads** | **Raw bases** | **Clean reads** | **Clean bases** | **Error rate (%)** | **Q20(%)** | **Q30(%)** | **GC content (%)** | **rRNA Ratio (%)** |
| --- | --- | --- | --- | --- | --- | --- | --- | --- | --- |
| DAQ-CA | 81041820 | 1.22E+10 | 80935290 | 1.19E+10 | 0.0283 | 96.66 | 91.18 | 47.03 | 4.87 |
| DAQ-ZC | 92360996 | 1.39E+10 | 92226040 | 1.35E+10 | 0.0255 | 97.8 | 93.62 | 46 | 5.1 |
| XHX-CA | 82513364 | 1.24E+10 | 82433692 | 1.2E+10 | 0.0257 | 97.72 | 93.44 | 45.92 | 5.38 |
| XHX-ZC | 77058566 | 1.16E+10 | 77016832 | 1.13E+10 | 0.026 | 97.63 | 93.21 | 45.46 | 5.21 |
| ZGZ-CA | 78589704 | 1.18E+10 | 78528026 | 1.15E+10 | 0.026 | 97.61 | 93.2 | 47.11 | 4.8 |
| ZGZ-ZC | 84432966 | 1.27E+10 | 84349184 | 1.23E+10 | 0.0258 | 97.68 | 93.36 | 47.18 | 6.9 |

Supplementary Table 2. lncRNA sequencing information of 3 paired CESC and non-tumor tissues.

CA: cancer tissue; ZC: control tissue.
